# 

**Published:** 1861-07

**Authors:** 


### CONTENTS.

ORIGINAL ESSAYS AND COMMUNICATIONS.

The Amalgam Question ........ 389
Diversities of Dental Practice. By J. Taft ........ 396

PROCEEDINGS OF SOCIETIES.

Indiana State Dental Association ........ 399

SELECTIONS.

A Mill for the Toothless ........ 402
Practical Hints ........ 403
Anæsthetic ........ 405
Dental Empiricism, Past and Present ........ 407
Diseases of the Mouth ........ 412
Chloroform ........ 418
Water: its History, Characteristics, Hygienic, and Therapeutic Uses ........ 421
Land Scurvey ........ 427
On a Method of Obtaining Homogeneous Light of Great Intensity ........ 428
Notices ........ 429
Editorial ........ 430

